# Gene set enrichment analysis of pathways and transcription factors associated with diabetic retinopathy using a microarray dataset

**DOI:** 10.3892/ijmm.2015.2220

**Published:** 2015-05-22

**Authors:** KAN HE, WENWEN LV, QING ZHANG, YUQING WANG, LIMING TAO, DAHAI LIU

**Affiliations:** 1 Center for Stem Cell and Translational Medicine, School of Life Sciences, Anhui University, Hefei, Anhui 230601, P.R. China; 2 Department of Ophthalmology, The Second Hospital of Anhui Medical University, Hefei, Anhui 230601, P.R. China

**Keywords:** diabetic retinopathy, pathway, gene set enrichment analysis, peroxisome proliferator-activated receptor, SMAD

## Abstract

Diabetic retinopathy (DR) is a serious microvascular complication of diabetes, which causes visual disability and blindness. Several studies have used gene expression profiling of DR to identify the key genes involved in this process; however, few studies have focused on the associated pathways and transcription factors (TFs), or on the co-expression patterns at the multiple pathways level. In this study, we employed a microarray dataset from the public database library of the Gene Expression Omnibus (GEO) associated with DR and applied gene set enrichment analysis (GSEA) to this dataset and performed candidate TF selection. As a result, 10 upregulated pathways, including the type I diabetes mellitus and peroxisome proliferator-activated receptor (PPAR) signaling pathways, as well as 59 downregulated pathways, including the ErbB signaling pathway and the mammalian target of rapamycin (mTOR) signaling pathway, were identified as DR-related pathways. The majority of these pathways have been previously identified, but some were novel. Finally, co-expression networks of related pathways were constructed using the significant core genes and TFs, such as PPARγ and SMAD4. The results of our study may enhance our understanding of the molecular mechanisms associated DR at the genome-wide level.

## Introduction

Diabetic retinopathy (DR), a specific microvascular complication of diabetes, is the most common cause of visual disability and blindness. The prevalence of DR increases with the duration of diabetes (1), and nearly 99% of patients with type 1 diabetes and 60% with type 2 have some degree of DR after 20 years (1,2). DR can be classified into 2 stages: non-proliferative and proliferative. The earliest visible signs of non-proliferative DR are microaneurysms, hemorrhages, hard exudates, cotton wool spots, intraretinal microvascular abnormalities and venous beading. The more severe state of proliferative DR (PDR) is characterized by the growth of new blood vessels on the surface of the retina or the optic disc, which are prone to hemorrhaging. Finally, visual impairment results in vitreous hemorrhage, subsequent fibrosis and tractional retinal detachment (3,4). Although the pathogenesis of DR has not yet been fully elucidated, the pathogenesis of diabetes is believed to be multifactorial, with genetic risk factors playing a fundamental role. However, several factors, including hyperglycemia, aldose reductase, advanced glycation end products (AGEs) and cytokines, such as vascular endothelial growth factor (VEGF) have been implicated in the disease pathogenesis (5).

Despite DR being a common complication of diabetes, little is known about the underlying molecular mechanisms. In recent years, the complex association that exists between the most relevant contributors to the onset and progression of DR, such as AGEs, oxidative stress, inflammation and angiogenesis have been elucidated and analyzed, particularly via whole-genome expression analyses using cells and animal models (6,7). Based on published studies, several systems, pathways and processes have been strongly implicated in DR; these include the renin-angiotensin system, the polyol pathway, non-enzymatic glycation, endothelial dysfunction, the maintenance of vascular tone, extracellular matrix remodeling and angiogenesis, which is dysregulated in diabetes and leads to the proliferation of new, fragile retinal capillaries and culminates in PDR (8,9). A host of genes involved in these pathways/processes have been treated as potential candidate genes. These genes include angiotensin I-converting enzyme (ACE), angiotensin II type 1 receptor (AGTR1), angiotensinogen (AGT), VEGF, aldose reductase (AR2), receptor for advanced glycation end products (RAGE), glucose transporter 1 (GLUT1), inducible nitric oxide synthase (NOS2A), constitutive nitric oxide synthase (NOS3), transforming growth factor-β (TGF-β), endothelin isoforms and their cellular receptors, amongst others (10–19). However, to the best of our knowledge, few studies to date have focused on the associated pathways and transcription factors (TFs), or on the co-expression patterns at the multiple pathways level.

In the present study, we employed a microarray dataset of genome-wide gene expression profiling from the Gene Expression Omnibus (GEO; http://www.ncbi.nlm.nih.gov/geo/) (20,21), which is associated with DR. The most well-known method of gene set enrichment analysis (GSEA) was used to analyze the genomic data in order to uncover the regulatory mechanisms of retinopathy (damage to the retina) caused by diabetic complications at the multiple pathways level. GSEA is widely used to analyze gene expression profiles, particularly to identify pre-defined gene sets which exhibit significant differences in expression between samples from the control and treatment groups (22–24). The goal of GSEA is to determine other inter esting categories (pathways) in which the constituent genes exhibit coordinated changes in expression under the given experimental conditions, other than in the form of sets of differentially expressed genes (DEGs). One of the advantages of GSEA is that it has the ability to highlight genes that are weakly connected to the phenotype through pathway analysis, something which may be difficult to detect using classical univariate statistics (22).

## Materials and methods

### Microarray data collection and pre-processing

We searched the GEO database(www.ncbi.nlm.nih.gov/geo/) for gene expression profiling studies associated with DR. Data were included in our re-analysis if they met the following conditions: i) the data were genome-wide; ii) a comparison was conducted between DR samples and control (CT) samples; and iii) complete microarray raw or normalized data were available. Finally, we chose the GSE12610 dataset for our re-analysis, which was contributed by Liang *et al* (http://www.ncbi.nlm.nih.gov/geo/query/acc.cgi?acc=GSE12610). In this dataset, a total of 5 RNA samples extracted from retinas were examined for RNA quality and then hybridized to 2 different GeneChip^®^ Mouse Genome 430 2.0 arrays (technical replicates; Affymetrix, Santa Clara, CA, USA). There were 3 biological replicates for DR (the samples from GSM315892 to GSM315894, designated as DR-1, DR-2 and DR-3) and 2 for CT (the samples GSM315895 and GSM315896, designated as CT-1 and CT-2).

In order to determine the influence of pre-processing on the comparison, data pre-processing was performed using software packages developed in version 2.6.0 of Bioconductor and R version 2.10.1. Each Affymetrix dataset was background adjusted, normalized, and log2 probe-set intensities were calculated using the Robust Multichip Average (RMA) algorithm from the affy package (25).

### GSEA

Our GSEA of pathways and genes was performed using the Category package in version 2.6.0 of Bioconductor (26). The goal of GSEA is to determine whether the members of a gene set S are randomly distributed throughout the entire reference gene list L or are primarily found at the top or bottom. One of the advantages of GSEA is its relative robustness in the face of noise and outliers in the data. In our analysis, the gene sets represented by <10 genes were excluded. The t-statistic mean of the genes was computed in each Kyoto Encyclopedia of Genes and Genomes (KEGG) pathway. Using a permutation test with 1,000 repetitions, the cut-off of significance level P-values was chosen as 0.05 for the significant pathways associated with DR. Accordingly, the significant pathways and genes were then identified by comparing the samples with DR and those with no DR. The following classification of identified pathways was based on the pathway maps br08901 of BRITE Functional Hierarchies in the KEGG database (http://www.genome.jp/kegg-bin/get_htext?br08901.keg). The annotation of significant genes in each pathway was performed using the biomaRt package, BioMart v 0.8 rc3 (version of 0.8 release candidate 3; http://www.biomart.org/). Next, clustering of groups and genes was performed based on the expression of the identified genes in each significant pathway, using the hierarchical clustering method and Pearson’s correlation co-efficient.

### Regulatory elements (REs) and TFs of co-regulated genes

We used a web server known as the DiRE (distant regulatory elements of co-expressed genes, http://dire.dcode.org/), which uses the Enhancer Identification (EI) method, to predict common REs for our input genes that have co-function in each identified, significantly related pathway (27). It predicts function-specific REs that consist of clusters of specifically associated transcription factor binding sites (TFBSs), and it also scores the association of individual TFs with the biological function shared by the group of input genes. We selected a random set of 5,000 genes in the genome of *Mus musculus* 9 (mm9) as the background genes. As a result, our predicted TFs have two major parameters, including TF occurrence (the percentage of candidate REs containing a conserved binding site for a particular TF) and TF importance (the product of TF occurrence and TF weight). From our candidate associated TFs with input gene sets, we selected the cut-off value of TF importance as >0.05.

## Results and Discussion

### Identification of significant pathways associated with DR

**Figure 1:**
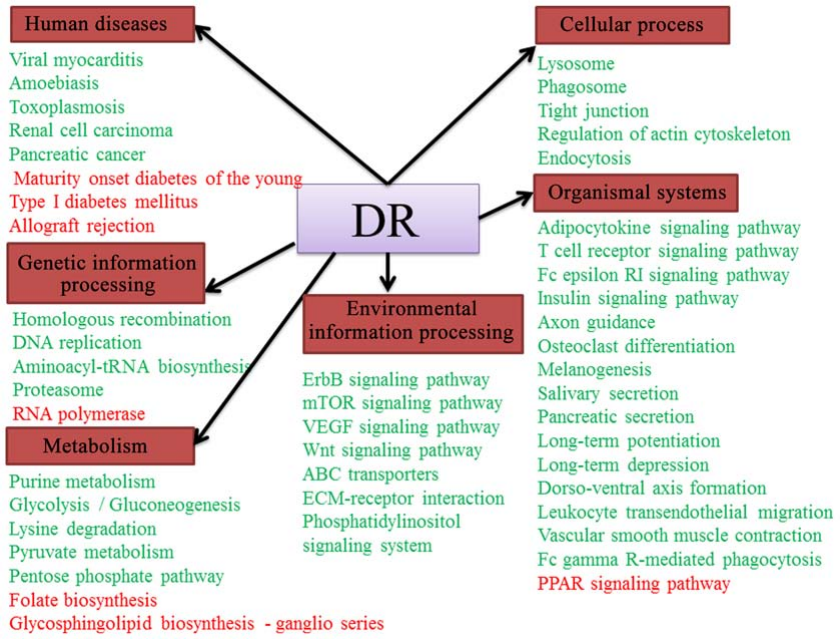
The coregulated pathways network related to diabetic retinopathy (DR) was established based on the 48 significant pathways identified by gene set enrichment analysis (GSEA). The names in red boxes represent 6 Kyoto Encyclopedia of Genes and Genomes (KEGG) pathway maps, and the names of significant pathways (upregulated pathways shown in red text and downregulated pathways shown in green) associated with DR.

**Figure 2:**
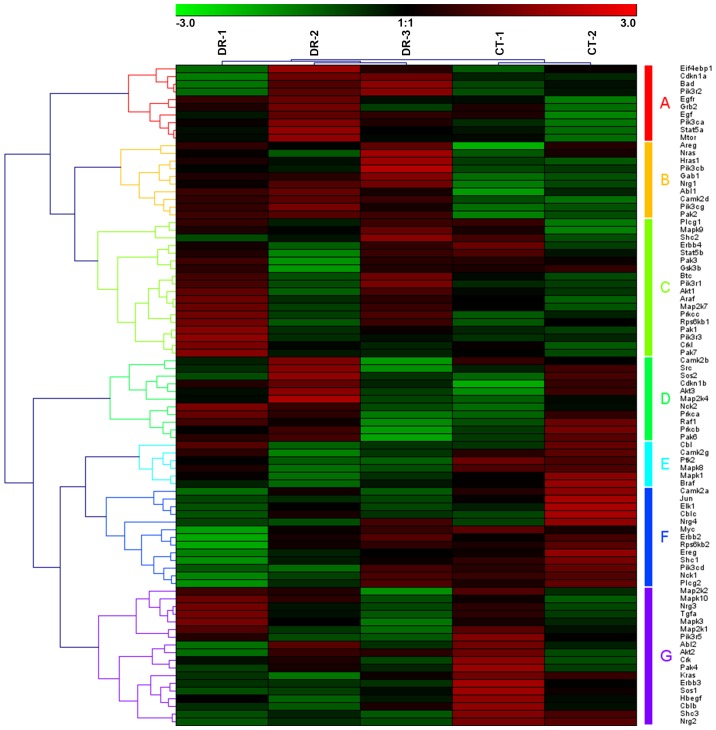
Heatmap of the downregulated ErbB signaling pathway was onstructed using the expression of 86 significant genes (probe sets), which may be clustered into 7 groups of gene sets based on hierarchical clustering with Pearson’s correlation co-efficient (group A-G). Five samples were clustered into 2 groups, diabetic retinopathy (DR)-1, DR-2 and DR-3 in one group and control (CT)-1 and CT-2 in the other.

**Figure 3:**
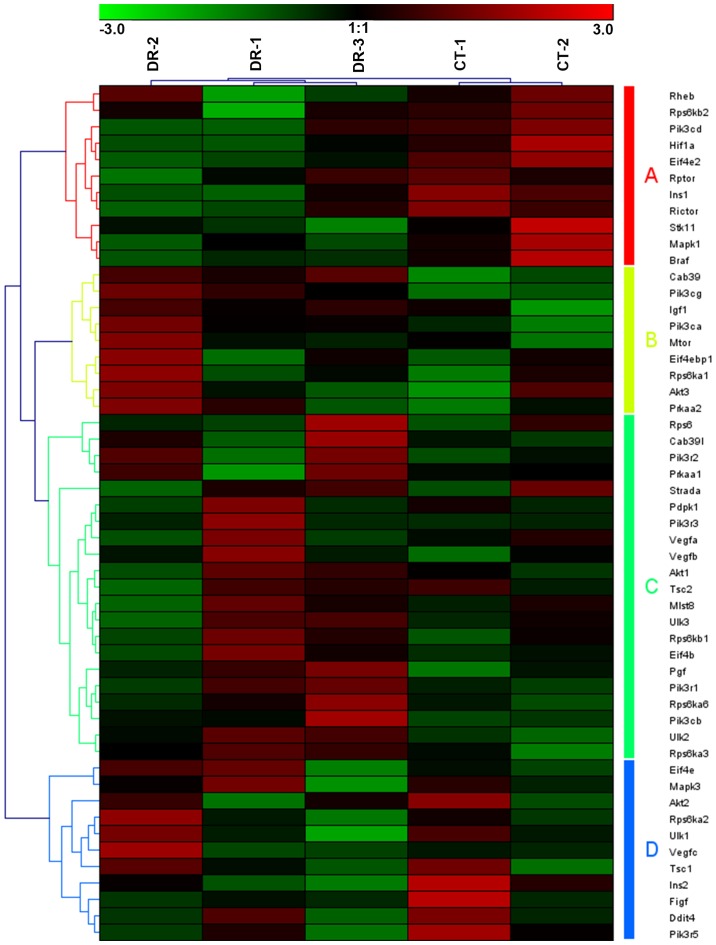
Heatmap of the downregulated of mTOR signaling pathway was constructed using the expression of 52 significant genes (probe sets), which may be clustered into 4 groups of gene sets based on hierarchical clustering with Pearson’s correlation co-efficient (group A-D). Five samples were grouped, with CT-1 and CT-2 in the control (CT) group and diabetic retinopathy (DR)-1, DR-2 and DR-3 in the DR group.

**Figure 4:**
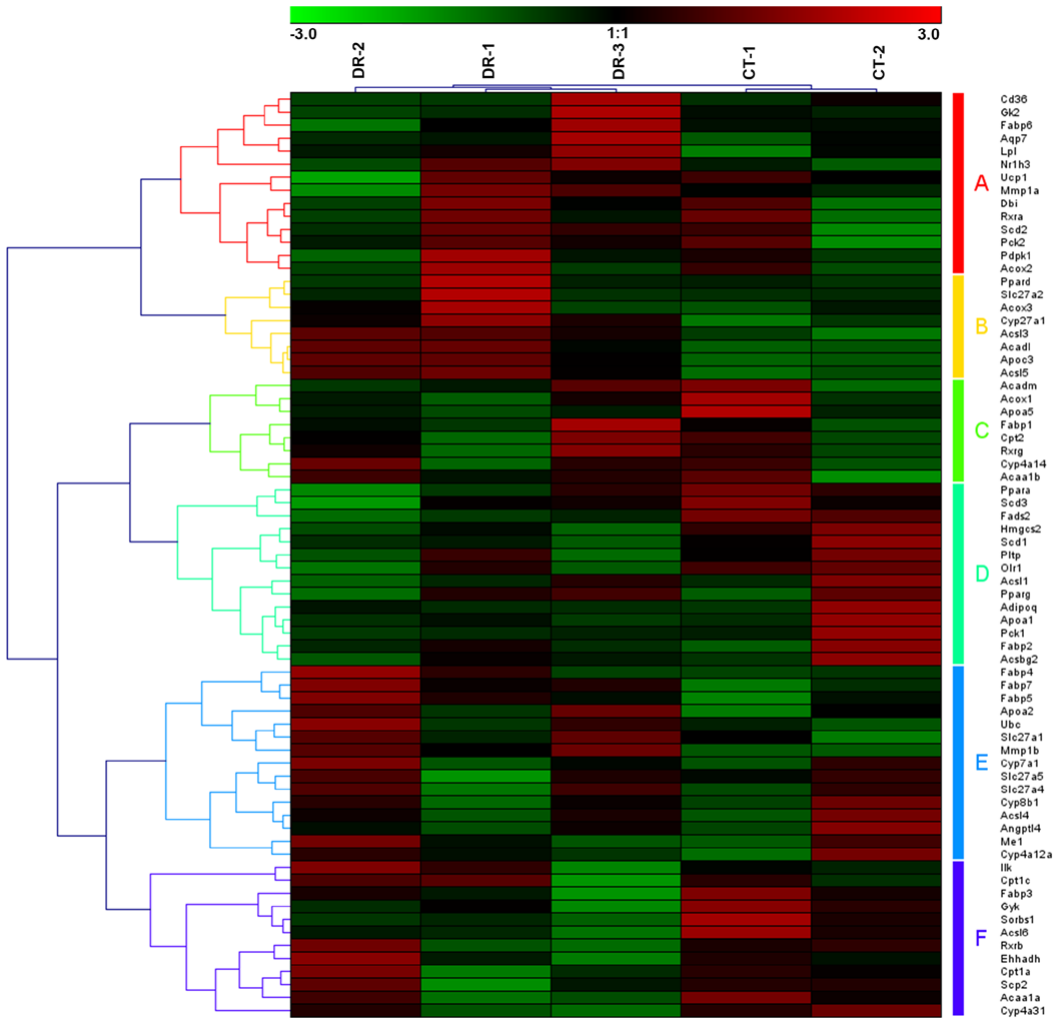
Heatmap of the upregulated peroxisome proliferator-activated receptor (PPAR) signaling pathway was constructed using the expression of 71 significant genes (probe sets), which may be clustered into 6 groups of gene sets based on hierarchical clustering with Pearson’s correlation co-efficient (group A–F). Five samples were clustered into 2 groups, with control (CT)-1 and CT-2 in one group and diabetic retinopathy (DR)-1, DR-2 and DR-3 in the other group.

Compared to the approach of DEGs, the strategy of GSEA that we used in this study is likely to be more powerful than conventional single-gene methods in the study of complex diseases, in which many genes make subtle contributions. According to our GSEA of the dataset of 5 samples, achieved by comparing the DR to the CT samples, there were 69 significant pathways associated with DR, whose P-values were <0.05, including 10 upregulated and 59 downregulated pathways. The coregulated pathways network is highlighted in Fig. 1 (red text indicates upregulated pathways, and green text indicates downregulated pathways). Furthermore, the details of significant genes in these 69 pathways related to DR are available upon request, as is the information on probe set ID and gene symbol. Among these 69 pathways associated with DR, the samples were classified and divided into DR and CT groups by clustering. For example, based on the expression of 86 significant genes whose significance level P-value was <0.05 in the downregulated ErbB signaling pathway, which may be clustered into 7 groups of gene sets (Fig. 2; group A-G), 5 samples were clustered into 2 groups, with DR-1, DR-2 and DR-3 in one group and CT-1 and CT-2 in the other group. Similarly, in the downregulated mammalian target of rapamycin (mTOR) pathway, 5 samples were also grouped, as CT-1 and CT-2 in the CT group and DR-1, DR-2 and DR-3 in the DR group (Fig. 3). The 52 genes involved in the mTOR signaling pathway may also be clustered into 4 groups of gene sets (Fig. 3, group A-D). Moreover, in the upregulated peroxisome proliferator-activated receptor (PPAR) pathway, 5 samples were also clustered into 2 groups, with CT-1 and CT-2 in one group and DR-1, DR-2 and DR-3 in the other group (Fig. 4). Furthermore, 71 genes were involved in the PPAR signaling pathway associated with DR, which may be clustered into 6 groups of gene sets (Fig. 4, group A–F). Moreover, based on the KEGG pathway maps in the KEGG database (http://www.genome.jp/kegg/), the 69 significant pathways could be mapped into 6 functional classes: cellular processes, environmental information processing, genetic information processing, human diseases, metabolism and organismal systems. The details of the pathways involved in each class are described in Tables I–V.

In the functional class of cellular processes, there were 6 significantly downregulated pathways related to DR (Table I). These pathways were involved in cell communication, cell motility, and transport and catabolism. Of these pathways, the tight junction one was the most significant, and was classified as belonging to the functional group of cell communication. As is well known, the breakdown of the blood-retinal barrier (BRB) is one of the most important characteristics of DR and is largely attributed to the disruption of endothelial tight junction. X-box binding protein 1 (XBP1), which is a major transcription factor activated by ER stress, plays an important role in maintaining endothelial barrier function (28). The activation of XBP1 protects against ER stress-induced tight junction damage.

There were 7 significantly downregulated pathways in the functional class of environmental information processing, 5 significantly downregulated pathways in the functional class of genetic information processing, and only 2 significantly upregulated pathways in the functional class of genetic information processing related to DR (Table II). The ATP-binding cassette (ABC) transporters pathway was associated with the function of membrane transport, and the extracellular matrix (ECM)-receptor interaction pathway was associated with the function of the signaling of molecules and interaction. The environmental information processing pathways, such as the ErbB, mTOR, VEGF and Wnt signaling pathways, and the phosphatidylinositol signaling system were related to the functions of signal transduction. The genetic information processing pathways, such as soluble N-ethylmaleimide-sensitive attachment protein receptor (SNARE) interactions in vesicular transport and proteasome were related to the functions of folding, sorting and degradation. The genetic information processing pathways of homologous recombination and DNA replication were related to the functions of replication and repair, and. Tthe genetic information processing pathways of aminoacyl-tRNA biosynthesis and ribosome were related to the functions of translation, and the RNA polymerase pathway was transcription-related. Of these aforementioned pathways, the ErbB and mTOR signaling pathways were the most significant in this class (the functional class of environmental and genetic information processing). The epidermal growth factor receptor (EGFR), also known as ErbB1/human epidermal growth factor receptor 1 (HER1), is a member of the ErbB family of receptor tyrosine kinases which also includes ErbB2 (Neu, HER2), ErbB3 (HER3) and ErbB4 (HER4). It was recently observed that hyperglycemia perturbs the EGFR-PI3K-AKT and extracellular signal-regulated kinase (ERK) signaling pathways in normal and healing corneas and that increased levels of cellular apoptosis and decreased cell proliferation may be contributing factors in the impairment of corneal epithelial wound healing in diabetic corneas (29,30). Signaling through the mTOR pathway plays a major role in smooth muscle and endothelial cell proliferation in response to hypoxia (31). There have been signs that the inhibition of the PI3K/Akt/mTOR pathway may have beneficial therapeutic effects in the management of PDR, which stems from findings that indicate that growth factors known to play major roles in the induction of angiogenesis depend on PI3K/Akt/mTOR for prolonging the cell survival signals that are operating in pathological angiogenesis (32).

Thirteen significantly associated pathways were classified and assigned to the functional class of human diseases, including 2 upregulated endocrine and metabolic diseases related pathways, 1 upregulated immune diseases related pathways, 3 downregulated cardiovascular diseases related pathways, 1 downregulated infectious diseases: bacterial related pathways, 4 downregulated infectious diseases: parasitic related pathways and 2 downregulated cancer related pathways (Table III). Of these, the pathway of type I diabetes mellitus was one of the most significant endocrine and metabolic diseases-related pathways. The prevalence of DR increases with the duration of diabetes. After 20 years of diabetes, nearly all patients with type I diabetes and >60% of patients with type II diabetes have some degree of retinopathy (33).

In the functional class of metabolism, there were 14 downregulated and 4 upregulated significant pathways associated with DR (Table IV). These were involved in 7 different types of metabolism, including carbohydrate metabolism, amino acid metabolism, nucleotide metabolism, lipid metabolism, metabolism of co-factors and vitamins, metabolism of terpenoids and polyketides, and glycan biosynthesis and metabolism. Of these, the glycolysis/gluconeogenesis pathway was one of the most significant pathways, which was classified and assigned to the functional group of carbohydrate metabolism. Protein kinase C (PKC) is a serine/threonine kinase, which is involved in signal transduction events with regard to specific hormonal, neuronal and growth factor stimuli. Hyperglycaemia leads to an increase in glucose flux through the glycolysis pathway, which in turn increases the *de novo* synthesis of diacylglycerol (DAG), the key activator of PKC in physiology (34). PKC is a molecule which plays an important role in the regulation of numerous physiological processes, whose upregulation contributes to the pathogenesis of DR.

In the last functional class of organismal systems, there was 1 significantly upregulated and 17 significantly downregulated pathways associated with DR (Table V). These were involved in the endocrine system, development, and the circulatory, excretory, digestive, nervous and immune systems. Of these, the PPAR signaling pathway was one of the most associated pathways, and was classified and assigned to the functional group of the endocrine system. PPARs are ligand-activated TFs (members of the nuclear receptor family) which offer promising targets for the development of novel, efficient treatments for several metabolic disorders. An indication suggesting that antidiabetic thiazolidinediones and adipogenic prostanoids are ligands of one of the PPARs reveals a novel signaling pathway that directly links these compounds to processes involved in glucose homeostasis and lipid metabolism, including adipocyte differentiation (35).

### Candidate TF selection associated with DR

To predict TFs potentially involved in the regulation of DR, we performed an analysis of TFBSs and predicted TFs using the significant genes in each identified pathway. Based on the cut-off value of TF importance, we identified the candidate TFs related to DR with potential target genes which are co-regulated in each of the above 69 pathways. The details are available upon request. As a result, 2 protein families, PPARs and SMADs, including members PPARα, PPARγ, PPAR_DR1, SMAD, SMAD3 and SMAD4, were predicted as candidate TFs in the majority of the identified pathways, particularly the downregulated pathways. PPARs are ligand-activated nuclear TFs that control gene expression by binding to specific response elements (PPREs) within promoters. They play a critical physiological role as lipid sensors and regulators of lipid metabolism (36). More potent synthetic PPAR ligands, including the fibrates and thiazolidinediones, have proven effective in the treatment of dyslipidemia and diabetes (35). The powerful therapeutic ability of PPARα and PPARγ agonists to favorably influence systemic lipid levels, glucose homeostasis, and the risk of atherosclerosis (in the case of PPARα activation in humans) have been demonstrated (37). PPARγ plays a vitally important role in the pathogenesis of DR by inhibiting retinal leukostasis and leakage in response to diabetes (38). Fenofibrate, a PPARα agonist, has demonstrated robust protective effects against DR in diabetic patients (39). Our data also support the hypothesis that PPARα and PPARγ may be important therapeutic targets for the management of DR.

The TGF-β signal is predominantly transduced by a family of TFs, the Smad proteins (40). After binding the TGF-β ligand from outside the cell surface, the type II receptor activates the type I receptor kinase, and this is followed by the phosphorylation of receptor-regulated Smads (R-Smads), Smad2 and Smad3. After associating with a common-partner Smad (co-Smad), or Smad4, the Smad complex translocates to the nucleus where it regulates the expression of target genes (40,41). TGF-β is a multifunctional cytokine with a number of biological effects, such as cell growth, differentiation and immunomodulation (42). A recent study found that the expression of TGF-β and Smad4 was increased in the retinal neovascular membrane of mice with oxygen-induced retinopathy (43). A previous study also indicated that increased Smad2/3 phosphorylation levels and increased TGF-β signaling do in fact occur in the retinal vessels of diabetic rats, and the concordant attenuation of such signaling by drugs which protect the vessels from the effects of diabetes through different mechanisms suggests that the increased signaling contributes to the vascular pathology (44).

In conclusion, we applied GSEA to re-analyze the published microarray datasets of DR and performed candidate TF selection. Finally, we identified 10 upregulated pathways, including the type I diabetes mellitus and PPAR signaling pathways, as well as 59 downregulated pathways, including the ErbB signaling pathway and the mTOR signaling pathway. Furthermore, co-expression networks of related pathways were constructed using the significant core genes and TFs, such as PPARG and SMAD4. These may be helpful to systematically understand the molecular mechanisms of diabetic retinopathy in genome-wide.

## Figures and Tables

**Table I:** Significant pathways associated with DR in the functional class of cellular processes.

Pathways	Map B	No. of genes	No. of TFs
04142: Lysosome	Transport and catabolism	119	28
04144: Endocytosis	Transport and catabolism	199	22
04145: Phagosome	Transport and catabolism	140	22
04530: Tight junction	Cell communication	127	34
04810: Regulation of actin cytoskeleton	Cell motility	206	26
05142: Chagas disease (American trypanosomiasis)		99	28

DR, diabetic retinopathy; TFs, transcription factors.

**Table II:** Significant pathways associated with DR in the functional class of environmental and genetic information processing.

Pathways	Map B	No. of genes	No. of TFs
02010: ABC transporters	Membrane transport	44	25
04512: ECM-receptor interaction	Signaling molecules and interaction	83	34
04012: ErbB signaling pathway	Signal transduction	86	40
04150: mTOR signaling pathway	Signal transduction	52	30
04370: VEGF signaling pathway	Signal transduction	74	25
04310: Wnt signaling pathway	Signal transduction	145	30
04070: Phosphatidylinositol signaling system	Signal transduction	73	26
04130: SNARE interactions in vesicular transport	Folding, sorting and degradation	35	6
03050: Proteasome	Folding, sorting and degradation	44	20
03440: Homologous recombination	Replication and repair	27	17
03030: DNA replication	Replication and repair	35	23
00970: Aminoacyl-tRNA biosynthesis	Translation	41	7
03010: Ribosome^a^	Translation	53	19
03020: RNA polymerase^a^	Transcription	24	29

a Significanlty upregulated pathways associated with DR. DR, diabetic retinopathy; TFs, transcription factors; ECM, extracellular matrix; VEGF, vascular endothelial growth factor; ABC, ATP-binding cassette; mTOR, mammalian target of rapamycin; SNARE, soluble N-ethylmaleimide-sensitive attachment protein receptor.

**Table III:** Significant pathways associated with DR in the functional class of human diseases.

Pathways	Map B	No. of genes	No. of TFs
04950: Maturity onset diabetes of the young	Endocrine and metabolic diseases	25	25
04940: Type I diabetes mellitus	Endocrine and metabolic diseases	40	30
05330: Allograft rejection	Immune diseases	33	22
05416: Viral myocarditis	Cardiovascular diseases	65	21
05410: Hypertrophic cardiomyopathy (HCM)	Cardiovascular diseases	81	32
05412: Arrhythmogenic right ventricular cardiomyopathy (ARVC)	Cardiovascular diseases	73	43
05100: Bacterial invasion of epithelial cells	Infectious diseases: bacterial	66	29
05140: Leishmaniasis	Infectious diseases: parasitic	62	20
05143: African trypanosomiasis	Infectious diseases: parasitic	30	19
05146: Amoebiasis	Infectious diseases: parasitic	106	26
05145: Toxoplasmosis	Infectious diseases: parasitic	123	19
05211: Renal cell carcinoma	Cancers	70	30
05212: Pancreatic cancer	Cancers	69	25

DR, diabetic retinopathy; TFs, transcription factors.

**Table IV:** Significant pathways associated with DR in the functional class of metabolism.

Pathways	Map B	No. of genes	No. of TFs
00010: Glycolysis/gluconeogenesis	Carbohydrate metabolism	57	14
00500: Starch and sucrose metabolism	Carbohydrate metabolism	32	12
00520: Amino sugar and nucleotide sugar metabolism	Carbohydrate metabolism	48	20
00620: Pyruvate metabolism	Carbohydrate metabolism	39	22
00030: Pentose phosphate pathway	Carbohydrate metabolism	25	14
00630: Glyoxylate and dicarboxylate metabolism	Carbohydrate metabolism	18	30
00250: Alanine, aspartate and glutamate metabolism	Amino acid metabolism	32	22
00290: Valine, leucine and isoleucine biosynthesis	Amino acid metabolism	11	30
00310: Lysine degradation	Amino acid metabolism	43	26
00230: Purine metabolism	Nucleotide metabolism	152	24
00590: Arachidonic acid metabolism	Lipid metabolism	77	21
00591: Linoleic acid metabolism	Lipid metabolism	40	13
00100: Steroid biosynthesis^a^	Lipid metabolism	18	17
00670: One carbon pool by folate	Metabolism of cofactors and vitamins	17	26
00790: Folate biosynthesis^a^	Metabolism of cofactors and vitamins	11	28
00900: Terpenoid backbone biosynthesis^a^	Metabolism of terpenoids and polyketides	13	22
00604: Glycosphingolipid biosynthesis-ganglio series^a^	Glycan biosynthesis and metabolism	15	19
00534: Glycosaminoglycan biosynthesis-heparan sulfate	Glycan biosynthesis and metabolism	24	25

a Significanlty upregulated pathways associated with DR. DR, diabetic retinopathy; TFs, transcription factors.

**Table V:** Significant pathways associated with DR in the functional class of organismal systems.

Pathways	Map B	No. of genes	No. of TFs
03320: PPAR signaling pathway^a^	Endocrine system	71	25
04910: Insulin signaling pathway	Endocrine system	128	27
04916: Melanogenesis	Endocrine system	95	40
04920: Adipocytokine signaling pathway	Endocrine system	67	19
04320: Dorso-ventral axis formation	Development	22	24
04360: Axon guidance	Development	129	28
04380: Osteoclast differentiation	Development	112	34
04270: Vascular smooth muscle contraction	Circulatory system	110	32
04960: Aldosterone-regulated sodium reabsorption	Excretory system	44	33
04962: Vasopressin-regulated water reabsorption	Excretory system	43	27
04970: Salivary secretion	Digestive system	70	41
04972: Pancreatic secretion	Digestive system	99	44
04720: Long-term potentiation	Nervous system	63	27
04730: Long-term depression	Nervous system	68	22
04660: T cell receptor signaling pathway	Immune system	106	33
04664: Fc epsilon RI signaling pathway	Immune system	77	28
04666: Fc gamma R-mediated phagocytosis	Immune system	86	39
04670: Leukocyte transendothelial migration	Immune system	115	26

a Significantly upregulated pathways associated with DR. DR, diabetic retinopathy; TFs, transcription factors; PPAR, peroxisome proliferator-activated receptor.
